# A Plea for Greater Accuracy in Our Therapeutics

**Published:** 1907-11

**Authors:** J. Robert Buchanan

**Affiliations:** Nevada, Mo.


### A PLEA FOR GREATER ACCURACY IN OUR THERAPEUTICS.*

By J. ROBT. BUCHANAN, M.D., Nevada, Mo.

In every department of the profession of medicine, the last quarter of a century has witnessed the most rapid strides and the most gigantic developments ever known. It is a matter of congratulation to the members of this grand profession to know that however great have been the developments in the sciences, the arts and mechanics, that the medical profession has kept

*Read before the Medical Association of the Southwest, Hot Springs, Ark., Oct. 8-10, 1907

step with them all, and to-day stands in the van of all progress.

Surgery in all her departments has achieved wonderful results. Obstetrics has advanced near to perfection. Biology has become the wonder of the age. Physiology has revealed to us the most intricate functions of the body. Chemistry, analytic and synthetic, has opened up to our vision the grandest secrets of nature. Pharmacy has joined hands with chemistry and materia medica and has erected manufactories for the preparation of the most scientific combinations of drugs, and on a scale undreamed of thirty years ago. Pathology, Histology and Bacteriology have laboriously delved among the diseased tissues of the body with scalpel and microscope, with test tube and reagent, until they have revealed to our astonished minds truths and conditions which had they been suggested a half century ago would have branded their advocate as a dreamer of the Ultra Utopian School. The Laboratory has come to stay. Hospitals and Dispensaries have been put under tribute and it would seem to the observer that nothing has been left undone to equip in the most perfect manner the modern doctor for a scientific discharge of his professional obligations.

Querry: In view of all the advancement, development and perfection of means to an end, mentioned above, is there any one department of this great structure that has in any way been overlooked?

What can we say of Therapeutics—the application of remedies for the relief of suffering? Never in the history of medicine has there been such a multiplication of remedies as within the last few years. It is the consensus of opinion of ninety per cent. of physicians who have practiced medicine for twenty-five or more years that the great majority of diseases can be successfully treated with a very limited number of remedies. If this be true, then what is the logical deduction to be drawn from the existence of the ten thousand and one remedies listed and described in our dispensatories. In many instances the same remedy is recommended for widely varying pathological conditions. In other instances innumerable remedies are recommended for the same pathological condition. It is not uncommon to find that in a given disease characterized by fixed pathological conditions we will observe that different authors or teachers in therapeutics will endorse or recommend the administration of drugs that are at absolute variance with each other from their physiologic or therapeutic effect. There seems to be but little, if any, unanimity of sentiment on the question of drug effect. This difference of opinion is not characteristic of the different schools only, but exists to an alarming extent among physicians of the same school.

This to my mind presents the most regretable phase of a divided profession. It is the "bete noir" of modern medicine. It constitutes the basis of the greatest source of distrust of the profession by the laity. How often have each of you observed this fact, that in a given case of surgery the only source of a difference of opinion between two or more surgeons is in arriving at a diagnosis of their case? After that, usually all is fair sailing. No controversy over the method of operating or over the technique to be adopted, there is a uniformity of opinion based upon definite teaching. Not so with the internist. Call two or more physicians to treat a case of pneumonia or typhoid fever, they may perfectly agree upon the diagnosis and the prognosis, but the chances are that if they do not disagree openly as to treatment they will at least have to each compromise to some degree in order to reach a middle ground upon which to base their treatment. It is not necessary that these physicians should represent different schools, as regular and eclectic, nor that they should be graduates of different colleges of the same school, as the college of Physicians and Surgeons, and the Medical department of University, but they may be and often are, graduates of the same college, taught by the same professors and are men of equal ability as observers and practitioners. Then why this difference of opinion? Why should Dr. A. recommend aconite, and Dr. B. reject it in a like case? Why should Dr. A. prefer the specific tincture of digitalis and Dr. B use only the fluid extract? Why should Dr. Jones say that calomel is only efficient in grain doses or more, while Dr. Smith never administers more than 1-10 gr. at a dose.

In other words, why should there not be a definite treatment for each special pathological condition when that condition affects a certain organ or system of organs? Why should not Catarrhal or lobar pneumonia, from a pathological viewpoint, be treated by the administration of the same remedies by Dr. Jones that are used by Dr. Smith? Why should typhoid fever, a disease now known to be the result of bacterial invasion, be treated by different methods and with such conflicting and opposing remedies by men of apparently equal ability and intelligence?

Why does Dr. C. assert positively that typhoid fever is a self-limited disease and that treatment can and does have no effect in arresting or abridging its course, while Dr. D. asserts that he can and has arrested, and even aborted the disease.

Why these conflicting opinions and opposing theories? In reply I will say that there are many reasons for this divided and uncertain condition that exists. Our profession is a developmental profession. It is not a science, neither in the true sense will it ever be. The elements entering into its makeup are necessarily such as will preclude absolute certainty on many points. It has to deal with a vital organism, that while in different individuals there exists a great similarity, there is also a great diversity; although built after the same model and endowed with the same vital element, with general characteristics similar, still there is sufficient dissimilarity or individuality to make each case as a law unto itself. The elements of personality will ever distinguish each individual from each other individual, and render a universal application of remedies an impossibility. Again, in the great struggle that has been going on in the profession to advance it to its present position we have in some way, to a degree, lost sight of the importance of practical therapeutics. We have not recognized the necessity of a closer study of therapeutic agents. We have been drifting in this branch of medicine. The preparation of our pharmaceutic agents has gradually dropped into the hands of commercialism.

The manufacturing pharmacist has to a great extent monopolized the field of pharmacology. He prepares our remedies for us; he makes the test of their strength; he indicates the doses, and often he suggests the character of the case in which the remedy is to be applied. One thing though he seldom if ever does, and that is to carefully test and properly label the amount or per cent. of active element contained in his preparation. He leaves the doctor to determine that by experiment on his patient. Here is where the profession necessarily drifts to sea. The doctor has absolutely no other means of determining the strength of these preparations except a clinical test. As there is fixed no uniform strength of these various remedies, tinctures, fluid extracts, etc., and as each manufacturer has his own standard of strength, if per chance he has a standard at all, and as it is the exception when any two samples of the same remedy, even when produced by the same manufacturer, contains a like per cent. of the alkaloid or active principle of the drug, then is it at all strange that physicians differ as to the advisability of using this or that drug in this or that form, or that they should become skeptical as to the use of drugs at all, and thus become therapeutic nihilists.

However great the debt of gratitude the physician owes the manufacturering pharmacist for the elegant and often efficient character of his preparations, still there is an obligation, imperative in its nature, resting upon the profession that they shall demand a uniformity of strength of the active elements contained in each sample of drug placed on the market by the manufacturer.

There should be a national law to this effect. The pure food and drug law should be so amended as to include this feature. In no other way is it possible to expect a uniformity of effect from the application of drugs, except they be of uniform strength.

As the therapeutic effect of a drug depends upon the amount of alkaloid or glucoside or active principle contained in it, and as a great number, even the great majority of our vegetable remedies are compound in their character, containing two or more active principles, and as these active principles are generally at variance with each other in their physiologic or therapeutic effect, and as one oten acts as a restraining or counter acting influence on the other; therefore it becomes the more important that there should be placed upon the label of each bottle or package of drugs that is offered to the profession, thus enabling the physician to know definitely what he is prescribing, and what he is to expect from its action.

This demand is slowly but surely formulating itself in the mind of the profession. We are gradually growing toward this assertion of a demand for a full and complete and correct statement of the strength of the drugs offered us. When this demand becomes more universal and imperative, then will come such a revolution in pharmaceutics and therapeutics as the average doctor has not dreamed of.

I see it coming and rejoice at its approach. It is not so far in the distance as many think, and when it arrives it will mark the most exact and satisfactory period in therapeutics that the profession has yet known.

When the pharmacist, by law is required to thus label each product he will then yield to the gradually growing demand that is springing up throughout the medical world to have all these active principles separated from each other, and from all superfluous extraneous matter, and presented to the physician in their purity and singleness thus enabling him to prescribe just what he needs and wants without having its action hindered and clogged by the presence of those principles that are not wanted or needed. Then will be given to therapeutics such an impetus as will stimulate an exactitude in prescribing and in result that will astonish both profession and laity. Thus do we hope for a realization of the title of this paper, "A Plea For Greater Accuracy In Our Therapeutics."

